# Abstracts and Gleanings

**Published:** 1888-11

**Authors:** 


### ABSTRACTS AND GLEANINGS.

**Calomel.**—The senseless opposition to calomel by Eclectics still goes on. The following remarks from (*Medical Brief*) by Dr. J. H. Hines, expresses our views: I have been practicing medicine for twenty years, in a malarious district, and started out very much opposed to calomel. I tried to substitute other remedies whenever I could, but in many cases, after losing precious time, have been compelled to resort to that *Magnum Dei bonum.* One of my especial friends, an M. D. of excellent reputation in our county, remarked to me a short time since, that if he had to exclude calomel and quinine from his saddle bags, he would hang them up to stay.

I have, in many cases, administered podophyllin, leptandrin, *et id omnegenus*, combined and re-combined, and failed most signally to produce bilious actions. But just as soon as I resorted to the use of calomel the secretions were aroused, and bilious dejections promptly followed, with relief to the patient and satisfaction to all concerned.

Theories, no matter how finely spun, will not stand against *facts*, which are the result of actual and often repeated clinical experiments; not on *dogs*, but experiments on man. Experiments on animals can not, for many obvious reasons, be fair. The bedside of a human being is the place to gather facts, and store them up for application in future cases.

Calomel *does* promote and increase the secretion of bile. Some persons are, of course, more sensitive to its action than others, but is this not the case with many other medicinal agents? Who of us has not met with individuals who could not bear quinine on account of some idiosyncrasy? Calomel has its place, and a very *large* place, in our armamentarium. I don't think it is going to be "voted out" soon. Its immoderate or injudicious use, no doubt has done, and will do, incalculable injury, but dose such misuse or abuse invalidate its medicinal virtues, when properly used? If so, the same can be said of *all* other medicinal agents, and the practice of our beloved profession is a failure.

Throw calomel aside? No, sirs; not as long as it yields such satisfactory results!

I believe that nine-tenths of the profession will take this side of the question, and their opinions are based on experience. While I would not accuse any of my brethren of insincerity or "indirection," I would not be surprised if some of *the other tenth* occasionally *slip in* a little mild chloride, when tackling a difficult case.

**Carbolic Acid in Hemorrhoids.**—"I have treated over two thousand cases of hemorrhoids by hypodermic injections of carbolic acid, and, although I do not use this method exclusively, I prefer it to any other. The patients are prepared for the operation as follows: For several days previous to it cathartics are taken, the last dose the night immediately previous. Just previous to the operation a large injection of slightly salt water is given. This washes out the rectum, brings down the 'piles,' and makes them accessible. The patient lies on a table, on the left side, with the knees we l drawn up to the abdomen. If the sphincter be much contracted, it is dilated with the forefinger This can be done without much pain, by the aid of cocaine. Nervous patients are anæsthetized with chloroform, which is preferred in such cases to ether. After thoroughly stretching the muscle, each 'pile' is injected with a solution of equal parts of olive oil and carbolic acid. The needle is introduced well into the tumor, since much more pain is occasioned by its simply passing under the mucous membrane than if it enter the body of the 'pile.' The whole 'pile' should slough out; which will not occur unless the injection be made into the tumor. The hemorrhoid is injected until it becomes milky-white. Each 'pile' is thus treated, and then returned into the rectum. For a moment or so there is a little smarting, after which all pain subsides. A few hours thereafter a hot, disagreeable, full sensation is felt. This hardly amounts to pain. In three or four days the tumor turns back and separates around the base. In about a week it has entirely sloughed off, and the base is partly healed In about two weeks the base has healed completely, and the patient is cured.

"Carbolic acid poisoning has not resulted from this procedure in my experience, nor has blood poisoning. Only two cases of extensive sloughing occurred and these in syphilitic subjects. There were six cases of easily controlled secondary hemorrhage. Several cases have suffered more than was agreeable, when tumor was not thoroughly injected. I have never lost a patient, nor failed to cure, when the tumor was properly injected. The treatment in my opinion, is a safe one, and yields excellent results."—*Dr. Monroe, in Medical Standard.*

**Hot Pack in Pneumonia.**—Dr. Carpenter (in *Medical Brief*) writes: Four years ago was attacked with pneumonia—on the left lung—dyspnœa severe, pain excruciating; friends all thought I must die from suffocation. One of my Homœopathic friends, a physician, called on me and gave the best consolation he could, by saying: "You know, doctor, few people ever recover at your age, from pneumonia." "Well, doctor," I said, "I am going to get well." I had myself placed in a reclining vapor-bath, and steam raised to about 110° to 130° F.; remained in it about two and a half hours; was free from pain and dyspnœa; began to cough and expectorate a little bloody sputa; was sick with fever; a few days, but by the use of hot water packs over and around my body an hour or two every day, and cathartics and quinine, I was able, in two weeks, to perform my usual amount of labor.

Again, two years ago, this winter, I was attacked with pneumonia of the right lung; about the same symptoms. Hepatization of nearly the whole lung took place. My Homœopathic brother and two Regulars, both good men, all volunteered to prescribe for me, like good men, as they were, and all thought and said it was very doubtf.l. I could not follow their treatment or advice—wou d immediately grow worse—and again resorted to the steam and hot pack, and in two weeks began to attend to business, and have not lost a day since.

My own, and other cases, have convinced me that pneumonia is a curable disease I have not lost a case in ten years, and do not expect to lose one in ten years to come, if I live to treat them. That it is a self-limiting disease I have no doubt, if left alone; but that it may be aborted at any stage I have no doubt, with judicious treatment; but never without first removing the cause and equalizing the circulation. Opening the pores of the skin and stimulating the capillary glands has been the sheet anchor of my hopes for years. And here let me remonstrate against dallying with so-called specifics, as you will surely lean on a broken staff. I believe there is science in the treatment of disease, and I hope the time will come when the profession will be able to see, eye to eye.

**A Valuable Formula in Menstrual Irregularities.**—Menorrhagia, metorrhagia, dysmenorrhea and amenorrhea are terms designating uterine conditions, common, yet often very intractable, exhausting the strength of the patience of the physician in their persistency.

If thoughtfully considered, these conditions are quite amenable to treatment in nearly every case.

Mrs. S., always regular in time and quantity of the menstrual flow, was obliged to do a prodigious amount of work in the care of her only sister to whom she was very much attached, and who had been taken suddenly and dangerously ill. The over-work, coupled with the anxiety and sorrow at the sudden death of the sister, brought on a metrorrhagia which seemed uncontrollable. The menses were excessive, amounting to flooding, continued from eight to ten days, then ceased for a week to return with increased severity. Ergot, erigeron, cinnamon, alum, gallic acid and other astringent measures did not produce an impression upon the disorder. The patient became exhausted and anemic. The following prescription was finally advised and the patient was kept quietly in bed for one week:

℞. Fl. ext. cenecio aurens........................
Fl. ext. aletris farinosa........................aa ℥ iij,
Fl. ext. cimicifuga racemosa.....................
Fl. ext. viburnum prunifolium.................aa ℥ v.

M. Sig.—Ten drops in water every three hours.

The flow soon ceased and did not return for a month. Then in proper quantity, and time has been very regular.

The above prescription seems to act more readily where the disturbance is of nervous origin, or is a result of nerve exhaustion.

In dysmenorrhœa, gelsemium has been added in place of cimicifuga, and very excellent results obtained. In amenorrhœa the prescription may be accompanied or alternated with iron in some form. In sub-involution, following confinement, the combination produces excellent results. In many cases it must be persisted in as it may not effect a cure immediately, but i, is generally prompt and satisfactory in its action. In old standing cases of "falling of the womb," the pain and distress, the backache and dragging sensations will be quickly relieved by this combination. The writer has used it for several years with excellent results and speaks from wide experience. The remedies are unpleasant of administration, but the smallness of the dose commends it to the physician.—*Chicago Medical Times.*

**Emergency Case.**—A writer in *Medical World* submits the following as a short list, and still one ready to meet any emergency:

1. Sulphate of morphia.
2. Sulphate of atropia.
3. Ergotine.
4. Tartar emetic.
5. Sulphate of zinc.
6. Dialyzed iron.
7. Carbonate of soda.
8. Tannic acid.
9. Chloroform.
10. Arom. spirits ammonia.
11. Chloral.
12. Croton oil.
14. Fl. extract of digitalis.

**Goitre.**—Dr. Chas A. Church, *in Medical World:*

℞. Ung. hydrag. iodide rubri (U. S. P.)
Adipis .................................... aa ʒ j,

M. et ft. unguentum. S.—Apply once or twice daily, until the skin is quite tender (not blistered), and keep it tender until the growth is removed.

In most cases the ointment alone is sufficient, but under cirtain circumstances, where there is a marked strumous diathesis, some form of iodine internally should be conjoined with the use of the ointment. I use the following prescription:

℞. Potassi iodidi .................................... ʒ j,

Syr. sarsaparillæ co .................................... f ℥ viij.

M. S.—Two teaspoonfuls before each meal.

I regard the above treatment as sure as any medical treatment can be, as I have never failed to either diminish the size of the goitre or remove it entirely.

**Diabetes—A New Drug in its Treatment.**—The syzygium jambolanum, or eugenia jambolana, is a new remedy recently introduced for the treatment of all cases of diabetes. It is found in India, Ceylon, Mauritium and Columbia. A tincture of the powdered seeds is used, in doses from 3 to 10 drops. It has been well tested in England, Germany, and in parts of the United States. It is said to prevent the formation of sugar in the system and to stay the waste.—*American Medical Journal.*

**Goitre—Gonorrhœa.**—Dr. Lowman (in *Medical Brief*) says: Goitre is most satisfactorily treated by means of electricity. Insert the needle of the negative pole into the tumor at the side, and place the positive pole on the opposite shoulder. The needle will be somewhat adherent after the use of the current for the correct length of time, which is about ten or fifteen minutes. Cells and detritus will have collected about the needle, but by reversing the poles for a short time, it will be readily extracted. Only a few cells are required, and a bi-weekly application of the current if desirable. This will, in a short time, greatly reduce the size of the tumor.

I have had great success in treating stricture by electricity. Recently had a case of deep stricture, which was an inch and a half in length, and through which no bougie could be passed. With a six-cell (McIntosh battery) galvanic current a normal sized bougie can be passed, and he does not suffer from any difficulty or retention of urine. He did not suffer any ill after effects, not even having the slightest urethral fever.

Gonorrhœa is also well treated by electricity, after the imflammatory stage is passed, the gonococci being effectually exterminated by the current.

In external hemorrh ids, I find the injection of normal liquid ergot to be the treatment *par excellence*. It acts like a charm in external piles and constant oozing. There is very little inflammation set up, due to the constringing effect of the ergot on the blood vessels. In a recent case, I injected 15 minims with no untoward symptoms, but with a perfect cure of the external, internal and oozing piles, from which the patient had been suffering ever since the late war.

I have also injected and administered ergot in ovarian fibromata with decided improvement.

**A Valuable Remedy for Chronic** Diarrhocea.—Many years ago I suffered from that trouble; I considered it incurable. Being in Paris, one of the best physicians there assured me it could be cured by a diet of racahout, and it was.

Afterward here I found one could not get the acorn meal that forms the active part, but knowing that its usefulness must depend on the tannin it contains, I tried substituting it as follows:

| | | |
|---|---|---|
| ℞. | Powdered chocolate, pure.......... .... | ½ ℔, |
| | Rice flour.............................. | ½ ℔, |
| | Powdered sugar........................ | ½ ℔, |
| | Tannin.................................. | ¼ oz. (120 grs.) |

The tannin, or the rest, separately, have little effect. Together they restore the tone of the alimentary canal and nourish as well as cure.

One thing is *essential*, that is, long cooking—not less than half an hour. If simply boiled a few minutes, the harsh taste of the tannin is very strong; with a good half hour's cooking, it disappears *wholly*—it is impossible to distinguish the medicine from ordinary broma. I think this has something to do with its curative powers, and with the ease of digestion by the most irritable stomach. The remedy is too valuable not to be more widely known.

The amount to be taken is a teacupful morning and evening at meals.—*Medical World.*

**Arrest of Hemorrhage from Wounds of the Palm of the Hand.**—(R. J. Levis, M. D., Philadelphia.)—My experience with hemorrhage from wounds of the palmar arches is, that it is usually controllable by maintaining extreme elevation of the hand. This is most thoroughly effected, and with the least discomfort to the patient, by vertical suspension of the limb, the attachment being made along the palmar and dorsal surfaces of the forearm by adhesive strips, after the ordinary manner of making extension in the treatment of fractures A cord from the adhesive straps may be fastened to the top of a bed-post or other convenient elevated point.

If posture alone should not arrest the hemorrhage, the most effective compression can be made by placing in the palm of the hand an India-rubber ball, or a ball solidly made of cotton wadding, and on this the fingers and thumb should be closed and bound tightly with a roller bandage.

Under these expedients, I have never been obliged to litigate arterial trunks for the arrest of hemorrhage from the palm of the hand.—*Medical and Surgical Reporter.*

**Aphrodisiac.**—Dr. Willard *in Medical Brief:* For an abiding stimulant to sexual desire and to make new blood (which, of course, depends more upon nutrition than drugs) I will append my favorite recipe for a life-rejuvenator—simple, harmless and positive—good and reliable as a blood maker:

℞. Hydrangeæ.............................℥ iv,

Acidi acet..................................℥ viij.

Agitate daily for eight days, then filter and add citrate of iron and phosphate of quinine 2 drachms. The dose is 6 drops in water, three times a day, after eating, increasing 1 drop each dose until you reach 20 drops to a dose.

**Battey vs. Lawson Tait.**—Lawson Tait in a note to the editor of the *N. Y. Medical Record* says: I have just seen, on p. 646, Vol. 33, of the *Medical Record*, the following remarkable statement: "Dr. Battey said that he had modestly, too modestly, remained quiet, and had allowed Mr. Tait to talk about 'his operation,' for he despised this miserable quibbling about priority."

If Dr. Battey is serious, why is he always raising the question and attacking me withont the slightest provocation? In the *Atlanta Medical Journal*, June, 1887, Dr. Battey made an equally intemperate attack on me, to which I replied in the March number of the same journal. Dr. Battey must have read my reply. Permit me to quote the last sentence of it as a reply to the assertion of Dr. Battey, quoted at the beginning of this letter: "It is far better and wiser not to use such meaningless, and therefore such confusing, phrases. I say there is no such thing as 'Tait's operation,' and—on this side of the Atlantic—there is many a poor wretch who wishes there had never been heard of such a procedure as 'Battey's.' "

I have more than once expressed a strong objection in your own columns to the phrase "Tait's operaton." I have never made any claim about an operation, and it is therefore diamettrically opposed to the truth when Dr. Battey says I have talked about "my operation." He ought to discuss the principle upon which we operate, and not the persons who do the operation, and if Dr. Battey would spend half the energy fighting those who oppose the progress of abdominal surgery that he directs against his friends, he might still live to do some great good in this world.

**External Application of Sulphur in Sciatic Neuralgia.**—A few years ago, in the Therapeutical Society of Paris, a discussion arose as to the best treatment of sciatica. Of course numerous methods were brought forward. The most novel, however, was one suggested by Dr. Henry Gneneau De Mussy. This treatment said Dr. Mussy, had been used in England with remarkable success.

The method simply consists in laying the affected limb or part in a bed of the flowers of sulphur, which was spread upon a cloth. How the sulphur acted was not known, but it was noticed that the urine was strongly odorous of sulphuretted hydrogen.

This treatment was followed by speedy relief, and, as a rule, the patient was entirely free from pain in less than twenty-four hours.

Dr. Henry Gneneau de Mussy told of a case where the valet of a certain ambassador had been seized with a most violent attack of neuralgia. On the following day the ambassador was to leave the city on a long journey, and was in great distress for fear that his servant could not accompany him. Dr. de Mussy having been called, immediately prescribed the external applicatian of flowers of sulphur, and on the following morning the recovery was complete, and the servant able to undertake the journey, to the great satifaction of his master.

Dr. L. Duchesne has recently adopted this treatment, and its use has always been attended with marked success.

In an article on the subject in the *Journal de Medicine*, Dr. Duchesne tells, among others, of the following case:

A lady, aged about 48, and of good constitution, had been for sometime past a most horrible sufferer from frequent and violent attacks of sciatica. She had tried innumerable remedies without ever finding any lasting relief.

Dr. Duchesne at once made an application of the flowers of sulphur to the affected parts. The limb was imbedded in the drug and covered with a cloth. In the morning, much to the patient's satisfaction, the neuralgia had entirely disappeared. Several years have elapsed, but there has never been a sign of the neuralgia's returning.

The treatment is both harmless and easy, and is apparently attended with the best results, and is therefore worthy of the attention of the profession.—*Therapeutic Gazette.*

**Antiseptic Method of Treating Burns and Scalds.**—Prof. S. W. Gross, of Philadelphia, suggests the following as by far the most efficient and painless method of managing burns and scalds. It is that practiced by Mosetig Moorhof, and it is the one invariably employed by Prof. Gross. The vesicles having been opened and excised, the entire burnt surface is smoothly covered with dry compresses of 20 per cent. iodoform gauze, over which gutta-percha is placed. The whole is then surrounded by a thick layer of sterilized absorbent cotton between layers of corrosive gauze, which is secured by a roller with a moderate degree of pressure. Such a dressing rapidly relieves pain, prevents contact of air and infection by septic pus, and by its permanence, keeps the parts at rest. It should be allowed to remain from seven to fourteen days. In burns of the second degree, one dressing suffices. In the worst burns, there is relatively little suppuration, and the eschars thrown off are aseptic. For burns of the face iodoform ointment (1 part iodoform, vaseline 20 parts), is used, and covered with a gutta-percha tissue mask. The ointment should be renewed daily.—*Practice.*

**Carbuncle.**—Dr. Main in *British Medical Journal* says, I have adopted the following for the last three years, to which I have added the hypodermic injection of cocaine. I inject into the carbuncle hypodermically half a grain of hydrochlorate of cocaine, and wait about five minutes until the skin is anesthetic; then I make a small incision in the center of the carbuncle with a tenotomy knife, and insert a small sharp piece of potassa fusa, and then push it home Afterward a piece of belladonna plaster is cut circular, a little larger than the carbuncle and placed over it. The plaster serves the double purpose of retaining caustic and alleviating the pain This is kept on for eight hours, and then it is taken off, and hot linseed poultices are applied for the same length of time. The result is that the patient always recovers about three days after the commencement of the treatment, which in this way is carried out almost painlessly.

**Diphtheria.**—Dr. Ady, *Medical and Surgical Reporter*, writes Having been a reader of medical literature, I tried every plausible plan of treating this terrible malady. I have come to the conclusion that remedies must be given with a view to their antiseptic properties. I generally commence with a decided mercurial cathartic, followed by antiseptics, mercurial or other, internally. Hydronaphthol is my favorite; it also keeps the house full of its fumes. Pilocarpine, sufficiently to keep the salvia flowing moderately; oil of turpentine internally in teaspoonful to tablespoonful doses, according to age; papoid or trypsin applied to the exudation, both in powder and spray; nux vomica as a nerve tonic, with all the food possible to get them to take, using, with the food, stimulants to aid assimilation. No swabbing or picking at the throat. I have lately seen a case of malignant diphtheria in a child seven years old recover under the above remedies, the turpentine being given in teaspoonful doses every two hours. I would not have given a penny for his chances when the treatment commenced the bronchial tubes being seriously obstructed.

**New Operation for Incontinence of Urine in Women.**—At the meeting of the British Gynecological Society, April 25, 1888, Dr. Willlam Alexander read a paper on this subject, in which he said that the first case he operated upon was a woman belonging to the theatrical profession, who often had to retain her uiine for unduly prolonged periods of time. This ultimately determined a paralysis of the sphincter of the urethra. To remedy the distressing effects of this condition of things, various methods of treatment were employed, but in vain, and ultimately he dissected out the uretha and led it into the rectum, hoping to utilize the rectal sphincier for the retention of urine. At the third attempt he succeeded. and the patient was much relieved. He also read the notes of two other cases on which he had operated for vesico-vaginal fistulæ by closing the vulva and carrying the urine into the rectum.—*Press and Circular.*

**Pilocarpine in Diphtheria.**—Dr. Lax has successfully treated a number of cases of diphtheria with pilocarpine. Under the influence of this drug the mucus and salivary secretions are greatly increased, large quantities of diphtheritic membrane are expelled from the throat and nose, respiration becomes more free. fever disappears, appetite returns, and convalescence is established in from three to five days. At the close of the attack every case manifested a characteristic eruption of herpes labialis.

The following formula was employed:—

℞. Pilocarpin. hydrochlorat............gr. $\frac{1}{3}$-3-5.

Pepsinæ..........................gr. x-xij

Acid. muriat dil......................*m* ij-iij.

Aquæ dest........................f℥xviiss. M.

Of this mixture from 1 to 4 teaspoonfuls were administered in wine, and warm fomentations were applied to the throat.

Guttman has treated in a year and a half eighty-one cases of diphtheria by pilocarpine, without the loss of a single patient. Gelsner and Dilewsky have also had good results from this treatment. The prescription given above may be varied as each case indicates, as also its dose.—*Journal de Medicine*, February 6, 1887.

**Strychnia in Dyspepsia.**—Flatulent dyspepsia frequently yields to the administration of sulphate of strychnia, combined with the interdiction of sugar, starchy foods, and beer, from the diet. A 20th part of a grain may be given three times a day. This form of dyspepsia is largely due to impaired action of the muscular coat of the stomach and deficient secretion; and strychnia is highly useful in both these conditions.—*Medical World.*

**The Treatment of Chronic Diarrhœa by Talc.**—The means at present employed in the treatment of chronic diarrhœas are as inadequate as they are numerous. Dr. Debove administered talc, in an impalpable powder, as being inert and not liable to undergo changes in contact with the intestinal secretions. He has given it in doses of seven to twenty ounces daily mixed with milk. In cases of diarrhœa due to tubercu'ous lesions of the intestines the treatment was uniformly successful, the diarrhœa being followed by an obstinate constipation. Curiously enough, he found that while giving the powdered talc patients tolerated substances which before they had been quite unable to retain. Milk, for example, which invariably gave rise to drastic purging, was given without setting up any disagreeable feelings. He has even been able to give a pound of oil in the twenty-four hours, and thus materially contribute to the nutrition of the patient. So far he has not tried the remedy in the diarrhœa of hot climates nor in children.—*Medical Age.*

**Hydrotherapy During Menstruation.**—At a discussion before the Societe d'Hydrologie, January, 1888 (*Gaz. de Gyn.*), upon the use of hydrotherapy during the catamenial period, the following conclusions were reached:

Recognizing the fact that applications of cold water during menstruation are sometimes followed by accidents; that these accidents are more frequent and serious with women suffering from pelvic diseases; that utero-ovarian diseases are often latent, and unknown to the patient, and, consequently, the statements of the patient are no guarantee of the pefect integrity of the pelvic organs:

1. The Society cannot sanction the continuance of hydrotherapy during the menstrual period in general practice.
2. It reserves the application of cold water for particular cases, in which there are urgent indications to be met—indications which it is difficult to meet in any other way.
3. In these exceptional cases, in the application of hydrotherapy, one must be sure of the condition of the uterus, and proceed only under the guidance of a competent physician.—*Medical Register.*

**Iodoform Poisoning.**—The absorption of iodoform gives rise to three distinct varieties of toxic symptoms, classified as the eruptive, the cerebral or delirious, and the syncopal forms. The eruptive form is the most common of the three. It is characterized by a reddish pimply eruption on various parts of the body, distant from the seat of absorption, and due probably to elimination of the poison by the cutaneous glands. In the cerebral form the patients may have epileptiform seizures or insomnia with delirium. The syncopal form is by far the most grave. Absorption is followed by a fall of temperature and algidity. In most cases the symptoms disappear on the discontinuance of the drug as a dressing, but it should in any event be employed with caution, as it is also liable to set up local irritation like any other powder.—*Medical Press and Circular.*

**A remarkable case** is reported by the *Bulletin Medicale* from Constantinople. Dr. Siotis had a patient who had swallowed fifteen pieces of gold. He complained of violent pains in the stomach. Upon auscultation one distinctly heard the rattling of the money. Some purgative medicine administered at once gave no result. He was then given pills of laudanum and belladonna. The next morning three pieces were found in the excrement, and a cylindrical tumor could be felt in the rectum, which was very painful. Four pieces were passed, and severe pain was felt in the iliac fossa, where percussion gave a metallic sound. The rest of the money soon followed, and the patient made a complete recovery.

The case is interesting from the quantity of the objects swallowed, and also from the fact that where purgatives were useless laudanum and belladonna gave the best results.—*Philadelphia Medical Times.*

**Acute Tonsilitis in Children.**—Dr. Frank Hamilton Potter says, in *Buff. Med. and Surg. Journal*:

1. When an inflammation attacks the tonsil, it is greatly influenced in its course by the presence of any diathesis.
2. The treatment must be so arranged as to meet and counteract the influence of this diathesis.
3. In all cases, simple as well as complicated, the general indications are to keep down the temperature and to relieve the local irritation.
4. The first indication can be met by the exhibition of antifebrin in proper doses; the second by the frequent application of bicarbonate of sodium, either in powder or in solution, to the surface of the tonsil.
5. This plan, properly followed, will generally limit the disease from one to three days.

**Digestive Disorders of Children.**—The value of listerine in those digestive disorders of childhood, which lead to what is commonly called cholera infantum, can scarcely be over-rated. A teaspoonful of listerine administered per orem has been known to dissipate the most alarming symptoms, cutting short the attack and apparently saving life. A good way is to begin something like this: Calomel and chlorate of potash each 1 grain, to be rubbed well together, and to be divided into ten powders; one to be given every five minutes until vomiting ceases, and the nature of the stools have been changed. Then commence and give teaspoonful doses of listerine every four hours until convalescence.—*Progress.*

**Dose of Aconitia—A Warning.**—The Pharmaceutical Society of Paris (*Presse med. Belge*, April 22, 1888, p. 135) sounds a note of warning in the use of *aconitia.* Numerous accidents from it were reported, though no notice of them had appeared in public print, and the alkaloid was declared perhaps the most violent poison known. Gr. 1-240 (the usual dose of Duquesnel's aconitia) had produced in an adult dangerous symptoms. Granules of this strength were condemned, and it was recommended to dispense those of a strength of gr. 1-600! in order rightly to proportion the dose. Digitalin was also declared powerful for evil and its dose too large.—*Amer. Jour. Med. Sciences.*

**Chronic Syphilitic Salivation.**—A. W. Furber, M. D., R. C. S. and L. D. S., says: I have for a long time had a—gentleman—patient under my care for disease of the teeth, and although my operations progressed favorably, I had many difficulties to contend with. The whole of my patient's teeth appeared to have a syphilitic taint, and with increased flow of saliva, amounting to chronic salivation. These were not the only troubles I had to surmount; but that which retarded my work most was the repeated recurrence of syphilitic ulcers of the sulcus and gums generally, which, though not painful to my patient, was still a source of considerable discomfort and militated greatly against the success of my operations. Iodia having come under my notice, I was inclined to give it a trial, and with the addition of a small proportion of liq. hydrarg. bi-chlor., taken daily before meals for a time—also occasionally as a mouth wash—the salivation became normal, the mucous membrane assumed a more healthy state and the teeth generally looked like coming back to their original color.

**Painless Destrucion of Nævia.**—A. B., aged two, suffering from a nævus the size of a shilling, behind the right ear, was on May, 13, 1887, treated by me in the following manner for its removal. Having first painted the healthy skin around the circumference of the nævus, for about half an inch, with a coating of collodion flexile, I applied a thick layer of a four per cent. solution of corrosive sublimate over the nævus. On the twenty-fifth, when I removed the collodion, the nævus had entirely disappeared, and nothing remained but a small scab. Dr. Boing was the first to suggest this method of treatment, and my object in publishing this case is to draw attention to so simple, satisfactory and painless method of treatment.—*British Medical Journal.*

**Use of Boracic Acid.**—It is well known that boracic acid is practically harmless. Gaucher has found it useful in impetigo, and the more so because it is without color or odor. The scabs should be removed by means of poultices, and a solution of boracic acid in glycerite of starch, 1-10, is then applied. Gaucher has cured a case of tuberculosis of the skin in the same way, and has given the acid in 10-grain doses in pulmonary tuberculosis, with advantage. The urine eliminates the acid readily and rapidly, and, as would be supposed, boracic acid is useful internally in cystitis, especially of old men.—*Lancet.*
—*Lancet.*

**A Vaginal Tampon.**—Dr. Frank Rattan, of Encinitas, Cal., writes: "The tampon as usually made has not been an entire success in my hands; it is rather difficult of removal by the patient.

I have made and used one of late that I think is an improvement on any with which I am familiar. Take an ounce package of ordinary sheet absorbent cotton It is about three and a half inches wide by half a yard long. Cut half in too, lengthwise, and sew, with a sewing machine, a seam down the middle. This holds the cotton together like a strip of cloth. It is easily applied and easily removed."

**Antipyrine as an Agalactic.**—Dr. Salemi, being called to a young robust woman, recently delivered of her first child, found it necessary to arrest the secretion of milk in consequence of the condition of the breasts. During ten days he tried all the usual means—purgatives, diet, iodide of potassium, etc, without effect. He then resorted to antipyrine, of which he gave 10 grains daily in three doses. The secretion diminished on the first day, and definitly on the third.—*Medical Age.*

**Transplantation of Skin from a Corpse.**—Dr. Bartens reports, in the *Berliner Klinische Wochenschrift* of August 6, 1888, a case of successful skin transplantation. The patient was a boy, aged fourteen, who was suffering from a loss of the integument of both feet consequent upon a burn. Some skin was taken from the legs of a man, aged seventy-five, who had died twenty minutes before, and was transplanted to the boy's feet. Cicatrization of the ulcers promptly followed.

**The best method** for curing fistula-in-ano according to Dr. Brinton, without the use of the knife, is by passing a silk or gum-elastic cord through the fistulous tract and bringing it out of the rectum and tying it. This will excite inflammation and the cord will gradually cut its way out followed by granulation. By this method the patient can be cured while following his ordinary occupation.—*College and Clinical Record.*

**Reduction of Paraphymosis.**—Paraphymosis may be easily reduced, it is said, by winding common twine firmly around the the organ, from the extremity backwards up to the middle of the member. In a few minutes, on removal of the twine, reduction can be quite easily effected by ordinary manipulation.—*Medical World.*

**Quassia in Drunkenness.**—A half ounce of ground quassia is steeped in an ounce of acetic acid, adding a pint of water before steeping. A teaspoonful in a little water should be taken every time the liquor thirst is felt. It satisfies the cravings and produces a feeling of stimulation and strength.—*N. E. Medical Monthly.*

**Methylal in Delirium Tremens.**—Dr. Krafft-Ebing asserts that he has obtained excellent results in the treatment of delirium tremens by the use of hypodermic injections of methylal. He says that it is indicated in cerebral anæmia, but is unfit for cases of cerebral excitement due to hyperæmia of the brain.

